# Complete mitochondrial genomes of two sand diver species (Perciformes, Trichonotidae): novel gene orders and phylogenetic position within Gobiiformes

**DOI:** 10.1080/23802359.2021.2005488

**Published:** 2021-12-10

**Authors:** Takashi P. Satoh, Eri Katayama

**Affiliations:** a The Kyoto University Museum, Kyoto, Japan; b Marine Stock-Enhancement Biology Laboratory, Graduate School of Agriculture, Kyoto University, Kyoto, Japan; c Research Institute of Marine Invertebrates, Tokyo, Japan; d Center for Collection, National Museum of Nature and Science, Tsukuba, Ibaraki, Japan

**Keywords:** Trichonotidae, Gobiiformes, mitogenome, gene rearrangements, phylogeny

## Abstract

The complete mitochondrial genome sequences of two species of the family Trichonotidae, *Trichonotus elegans* (Shimada and Yoshino 1984) and *Trichonotus filamentosus* (Steindachner 1867), were determined using a polymerase chain reaction-based method. The genomes ranged from 16,517 to 17,235 bp in length and included 37 genes (13 protein-coding genes, 22 transfer RNA genes, and 2 ribosomal RNA genes) and two non-coding regions (control region and origin of the light strand replication) as in other vertebrates. However, they shared a unique gene order among vertebrates with multiple gene switching and insertions. Phylogenetic analysis showed that Trichonotidae and Apogonidae are sister groups, which together with Kurtidae are placed as a closely related clade of Gobioidei. These results would be useful for analyzing the evolutionary relationships of Gobiiformes and the evolutionary study of fish mitogenomes.

Trichonotidae (sand divers) is a small monogeneric family comprising 10 valid species (Nelson et al. 2016), all of which are distributed in the Indo-Pacific region. Sand divers have an elongated, cylindrical body, a pointed snout, and long-based dorsal and anal fins. They are found on the sandy sea bottoms at about 10–30 m depths, quickly dive into sand, and bury themselves when threatened. Although their commercial value is not high, they are popular as ornamental fish for scuba divers. Recently, the existence of an undescribed species and a geographical variation related to Trichonotidae have been suggested (Kuiter and Tonozuka 2004; Katayama and Endo 2010), requiring detailed taxonomic studies, including molecular analysis. However, genetic information on trichonotids is insufficient, as only nucleic or partial mitochondrial genome (mitogenome) sequences of ∼1000 bp have been registered in public databases. This study determined the complete mitogenome sequences of two trichonotids (*Trichonotus elegans* and *Trichonotus filamentosus*) and conducted a phylogenetic analysis, including Gobioidei, which are closely related to *Trichonotus* based on previous studies (Thacker et al. 2015; Betancur et al. 2017).

*T. elegans* specimen was purchased from an ornamental fish store and deposited at the Natural History Museum and Institute, Chiba (accession no. CBM-ZF 10754: curator Masaki Miya, miya@chiba-muse.or.jp). *T. filamentosus* specimen was collected by sledge net off Tosa Bay (33.355928 N, 133.497122 E) and deposited at Kochi University (accession no. BSKU 95895: curator Hiromitsu Endo, endoh@kochi-u.ac.jp). These specimens were identified based on each original description (Steindachner 1867; Shimada and Yoshino 1984). Total genomic DNA was extracted from muscle tissue using the Gentra Puregene Tissue Kit (Qiagen) according to the manufacturer’s protocol. The complete mitogenomes of the two specimens were amplified and sequenced using a combination of long and short polymerase chain reactions (PCRs) and direct cycle sequencing techniques (Miya and Nishida 1999). These reactions were carried out as previously described (Miya and Nishida 1999; Satoh et al. 2016). The mitogenomes were 16,517 bp (*T. elegans*: GenBank accession no. AP006817) and 17,235 bp (*T. filamentosus*: GenBank accession no. AP018348) in length, containing 13 protein-coding genes, 2 ribosomal RNA (rRNA) genes, 22 transfer RNA (tRNA) genes, and a control region, as found in other vertebrates. Also, most genes were encoded on the H-strand, except for the ND6 and eight tRNA genes, all genes being similar in length to those in other vertebrates. The base compositions of the two species were A (25.58%), C (28.76%), G (19.16%), and T (26.50%) for *T. elegans* and A (26.47%), C (28.51%), G (18.11%), and T (26.90%) for *T. filamentosus*, which were similar to those observed in most other fishes (Satoh et al. 2016).

The mitogenomes of both species shared deviations from the highly conserved gene order in vertebrates (gene rearrangements), such as gene switching in two regions (tRNA-Pro gene and control region, tRNA-Asn gene, and origin of L-strand replication). These characteristic gene orders were shown to be a molecular marker supporting the monophyly of Trichonotidae. Furthermore, *T. filamentosus* had a unique insertion sequence (632 bp) between COIII and tRNA-Gly genes, representing a remnant of incomplete deletions from the putative duplicated region. Additionally, *Kurtus gulliveri*, included in the analysis as a member of the order Gobiiformes, also had a unique gene rearrangement, such as switching of tRNA-Ile and tRNA-Gln genes. Miya et al. (2013) first published the whole mitogenome sequence from this species, but they did not mention the gene rearrangement of this species.

To confirm the phylogenetic position of Trichonotidae, the complete mitogenome sequences of 25 species, mainly from Gobiiformes, which are closely related to trichonotids in previous studies (Thacker et al. 2015; Betancur et al. 2017), were retrieved from GenBank. Maximum likelihood (ML) phylogenetic analyses were conducted using mitochondrial 12 protein-coding genes. The details of the analysis method are described in Figure 1. The resultant ML tree strongly supported the monophyly of Gobiiformes and indicated that trichonotids were most closely related to Apogonidae (Figure 1). In this study, the phylogenetic relationships among families within Gobiiformes differed from the topology of Thacker et al. (2015) and McCraney et al. (2020), which showed a sister grouping of Trichonotidae and Gobioidei, but were not strongly supported in the bootstrap analysis (Figure 1). Mitogenomes with gene rearrangements, such as *Trichonotus* and *Kurtus*, tend to have high nucleotide substitution rates (Dowton and Austin 1999; Shao et al. 2003). Although apogonids did not have gene rearrangements, their evolutionary rate seemed much faster than Gobioidei. Therefore, it is possible that this resultant tree was affected by long-branch attraction (Bergsten 2005). To obtain more accurate phylogenetic relationships for this group, it would be necessary to expand *Trichonotus* and *Kurtus* data and conduct analyses with appropriate corrections.

**Figure 1.**
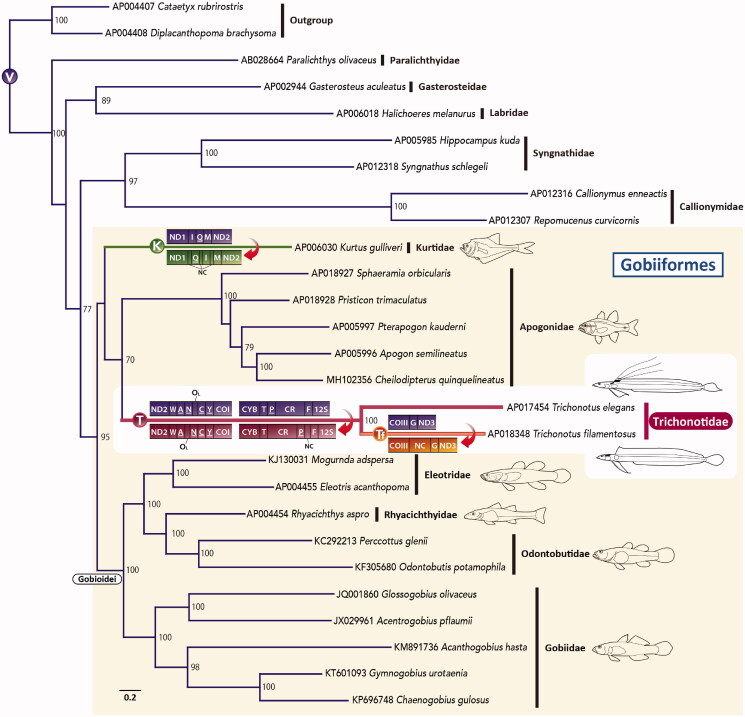
An ML phylogenetic tree was inferred using the program RAxML (Stamatakis 2014) and the timing of gene rearrangements in each mitogenome. Accession numbers are indicated before the species names. Bootstrap values above 50% were shown at each node. The analysis was conducted on a data matrix (10,767 positions), including the concatenated nucleotide sequences of protein-coding genes in the mitogenomes, except the *ND6* gene. Gene sequences were aligned individually using the online version of MAFFT (http://mafft.cbrc.jp/alignment/server/. ; Katoh and Standley 2013), and ambiguous regions were trimmed using the online version of Gblocks with the least stringent settings (http://molevol.cmima.csic.es/castresana/Gblocks_server.html. ; Castresana 2000). The optimal partition model was determined using PartitionFinder version 2 (Lanfear et al. 2017). Rapid bootstrap analyses were conducted with 1000 replications. Partitioned ML analyses were performed with RAxML-GUI version 2.0.1 (Edler et al. 2021) using the GTRGAMMAI nucleotide substitution model. Circled V, K, T, and Tf represent the gene order of typical vertebrates, *Kurtus*, trichonotids, and *T. filamentous*, respectively. tRNA genes are designated by single-letter amino acid codes. 12S, 12S rRNA gene; COI and COIII, cytochrome c oxidase subunits I and III genes, respectively; CR, putative control region; Cyt b, cytochrome b gene; NC, noncoding sequences; ND1–3, NADH dehydrogenase subunit 1–3 genes, respectively; O_L_, the origin of L-strand replication.

## Data Availability

The mitochondrion genome sequence data that support the findings of this study are openly available in GenBank of the National Center for Biotechnology Information (NCBI) at https://www.ncbi.nlm.nih.gov/ under the accession nos. AP006817 and AP018348.

## References

[CIT0001] Bergsten J. 2005. A review of long-branch attraction. Cladistics. 21(2):163–193. 34892859 10.1111/j.1096-0031.2005.00059.x

[CIT0002] Betancur RR, Wiley EO, Arratia G, Acero A, Bailly N, Miya M, Lecointre G, Ortí G. 2017. Phylogenetic classification of bony fishes. BMC Evol Biol. 17(1):162. 28683774 10.1186/s12862-017-0958-3 PMC5501477

[CIT0003] Castresana J. 2000. Selection of conserved blocks from multiple alignments for their use in phylogenetic analysis. Mol Biol Evol. 17(4):540–552. 10742046 10.1093/oxfordjournals.molbev.a026334

[CIT0004] Dowton M, Austin AD. 1999. Evolutionary dynamics of a mitochondrial rearrangement "hot spot" in the hymenoptera. Mol Biol Evol. 16(2):298–309. 10028295 10.1093/oxfordjournals.molbev.a026111

[CIT0005] Edler D, Klein J, Antonelli A, Silvestro D. 2021. raxmlGUI 2.0: a graphical interface and toolkit for phylogenetic analyses using RAxML. Methods Ecol. Evol. 12:373–377.

[CIT0006] Katayama E, Endo H. 2010. Redescription of a Sanddiver, *Trichonotus blochii* (Actinopterygii: Perciformes: Trichonotidae), with confirmation of its validity. SpecDiv. 15(1):1–10.

[CIT0007] Kuiter RH, Tonozuka T. 2004. Pictorial guide to: Indonesian reef fishes, part 2, 2nd ed., PT Dive & Dive’s, Bali, Denpasar.

[CIT0008] Katoh K, Standley DM. 2013. MAFFT multiple sequence alignment software version 7: improvements in performance and usability. Mol Biol Evol. 30(4):772–780. 23329690 10.1093/molbev/mst010 PMC3603318

[CIT0009] Lanfear R, Frandsen PB, Wright AM, Senfeld T, Calcott B. 2017. PartitionFinder 2: new methods for selecting partitioned models of evolution for molecular and morphological phylogenetic analyses. Mol Biol Evol. 34(3):772–773. 28013191 10.1093/molbev/msw260

[CIT0010] McCraney WT, Thacker CE, Alfaro ME. 2020. Supermatrix phylogeny resolves goby lineages and reveals unstable root of Gobiaria. Mol Phylogenet Evol. 151:106862. 32473335 10.1016/j.ympev.2020.106862

[CIT0011] Miya M, Nishida M. 1999. Organization of the mitochondrial genome of a deep-sea fish, *Gonostoma gracile* (Teleostei: Stomiiformes): first example of transfer RNA gene rearrangements in bony fishes. Mar Biotechnol. 1(5):416–426. 10.1007/pl00011798 10525676

[CIT0012] Miya M, Friedman M, Satoh TP, Takeshima H, Sado T, Iwasaki W, Yamanoue Y, Nakatani M, Mabuchi K, Inoue JG, et al. 2013. Evolutionary origin of the scombridae (tunas and mackerels): members of a paleogene adaptive radiation with 14 other pelagic fish families. PLOS One. 8(9):e73535. 24023883 10.1371/journal.pone.0073535 PMC3762723

[CIT0013] Nelson JS, Grande TC, Wilson MVH. 2016. Fishes of the world. 5th ed. Hoboken, New Jersey: John Wiley & Sons, Inc.

[CIT0014] Satoh TP, Miya M, Mabuchi K, Nishida M. 2016. Structure and variation of the mitochondrial genome of fishes. BMC Genomics. 17:719. 27604148 10.1186/s12864-016-3054-y PMC5015259

[CIT18216937] Shao R. Dowton M, Murrell A, Barker SC. 2003. Rates of Gene Rearrangement and Nucleotide Substitution Are Correlated in the Mitochondrial Genomes of Insects. Mol. Biol. Evol. 20(10):1612–1619. doi:10.1093/molbev/msg176. 12832626

[CIT0015] Shimada K, Yoshino T. 1984. A new trichonotid fish from the Yaeyama Islands, Okinawa Prefecture, Japan. Jpn J Ichthyol. 31:5–1.

[CIT0016] Stamatakis A. 2014. RAxML version 8: a tool for phylogenetic analysis and post-analysis of large phylogenies. Bioinformatics. 30(9):1312–1313. 24451623 10.1093/bioinformatics/btu033 PMC3998144

[CIT0017] Steindachner F. 1867. Icththyologische Notizen (V). Sitzungsberichte Der Kaiserlichen Akademie Der Wissenchaften-Naturwissenschaftliche Class. 55:701–716.

[CIT0018] Thacker CE, Satoh TP, Katayama E, Harrington RC, Eytan RI, Near TJ. 2015. Molecular phylogeny of Percomorpha resolves *Trichonotus* as the sister lineage to Gobioidei (Teleostei: Gobiiformes) and confirms the polyphyly of Trachinoidei. Mol Phylogenet Evol. 93:172–179. 26265255 10.1016/j.ympev.2015.08.001

